# JSI-124 Induces Cell Cycle Arrest and Regulates the Apoptosis in Glioblastoma Cells

**DOI:** 10.3390/biomedicines11112999

**Published:** 2023-11-08

**Authors:** Tai-Hsin Tsai, Kuan-Ting Lee, Yi-Chiang Hsu

**Affiliations:** 1 Department of Surgery, School of Medicine, College of Medicine, Kaohsiung Medical University, Kaohsiung 807378, Taiwan; tsaitaihsin910077@gmail.com; 2 Division of Neurosurgery, Department of Surgery, Kaohsiung Medical University Hospital, Kaohsiung 807378, Taiwan; 3 Graduate Institutes of Medicine, College of Medicine, Kaohsiung Medical University, Kaohsiung 807378, Taiwan; ayta860404@gmail.com; 4 Division of Neurosurgery, Department of Surgery, Kaohsiung Municipal Ta-Tung Hospital, Kaohsiung 80145, Taiwan; 5 School of Medicine, I-Shou University, Kaohsiung 82445, Taiwan

**Keywords:** cucurbitacin I, glioblastoma, cell cycle, apoptosis

## Abstract

Cucurbitacin I (JSI-124), derived from Cucurbitaceae, has shown the potential to induce apoptosis and cell cycle arrest in some cancer cells. However, the effect of JSI-124 on glioblastoma multiforme (GBM) cell cycle and apoptosis is still unclear. Our investigation revealed that JSI-124 effectively reduced cell viability in GBM cells, leading to apoptosis and increased caspase-3 activity. Intriguingly, JSI-124 caused the accumulation of G2/M phase to regulate cell cycle, confirmed by MPM-2 staining and increased protein synthesis during mitosis by mitotic index analysis. Western blot analysis found that JSI-124 affected the progression of G2/M arrest by downregulating the CDK1 and upregulating the cyclinB1, suggesting that JSI-124 disrupted the formation and function of the cyclin B1/CDK1 complex in GBM8401 and U87MG cells. However, we found the JSI-124-regulated cell cycle G2/M and apoptosis-relative gene in GBM8401 and U87MG cells by NGS data analysis. Notably, we found that the GBM8401 and U87MG cells observed regulation of apoptosis and cell-cycle-related signaling pathways. Taken together, JSI-124 exhibited the ability to induce G2/M arrest, effectively arresting the cell cycle at critical stages. This arrest is accompanied by the initiation of apoptosis, highlighting the dual mechanism of action of JSI-124. Collectively, our findings emphasize that JSI-124 holds potential as a therapeutic agent for GBM by impeding cell cycle progression, inhibiting cell proliferation, and promoting apoptosis. As demonstrated by our in vitro experiments, these effects are mediated through modulation of key molecular targets.

## 1. Introduction

In spite of the advancements achieved through multidisciplinary treatment approaches, glioblastoma multiforme (GBM) still persists as a profoundly fatal brain tumor with a dismal overall survival rate in the adult population. At present, the median survival for GBM stands at a mere 12.6 months [1]. The standard treatment for GBM involves extensive surgical resection, along with the incorporation of chemotherapeutic strategies and radiation therapy in this multidisciplinary approach. Despite significant progress through this combination of therapies, improvements in patient survival remain limited, often resulting in poor survival, high relapse rates, and treatment resistance in GBM [2,3]. With the continuous advancement of research on the genetic underpinnings of glioblastoma, there is a hopeful anticipation that novel biomarkers and therapeutic targets will emerge. This holds the potential to unlock new opportunities for effective treatment strategies.

**Figure 1:**
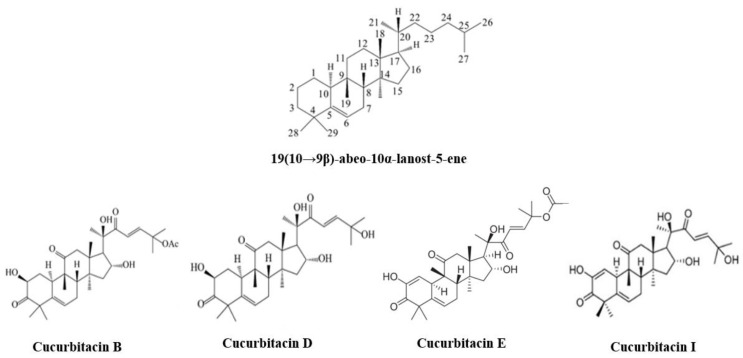
Structure of Cucurbitacins family.

Nutraceuticals, derived from plant extracts such as Cucurbitacin and *Urtica dioica* [4], are playing an increasingly significant role in modern life. They contribute to the strengthening of the immune system, alleviation of joint pain, enhancement of heart health, improvement of cognitive function, and even to the prevention and treatment of cancer [4,5,6]. Cucurbitacin I (JSI-124) belongs to the Cucurbitacin family of drugs. It is a naturally occurring tetracyclic triterpenoid compound [7]. The Cucurbitacins are characterized by a chemical formula diagram isolated from that of the Cucurbitaceae family (including Cucurbitacin B, D, E, and I), as shown in (Figure 1). It is derived from plants within the Cucurbitaceae family including Cucurbitaceae and Brassicaceae. In traditional medicine, cucurbitacins have been utilized for their anti-inflammatory and analgesic properties [8]. In addition, JSI-124 has been shown to be a strong activator of apoptosis in multiple tumor cell lines (e.g., GNM) and to be implicated in the effects of other antineoplastic drugs [9].

Moreover, research has shown that JSI-124 is a selective inhibitor of JAK2/STAT3 and also induces non-selective activation of the JNK/c-Jun pathway while disrupting the actin cytoskeleton [10,11,12]. Previous studies have provided evidence of JSI-124 effectively inhibiting the growth of various malignant tumors such as lung cancer, breast cancer, B leukemia, and gastric cancer [10,12,13,14]. Although the current literature consistently indicates that cucurbitacin I (JSI-124) possesses characteristics indicative of inhibition of cell proliferation and anti-tumor effects, there is currently no supporting evidence for glioblastoma in any literature.

Accumulating evidence has shown that JSI-124 has the ability to induce apoptosis and autophagy, while causing cell cycle arrest in glioblastoma cells. However, the use of Chinese herbal medicines for treatment has been widespread for many years, suggesting a potential common mechanism of action and further supporting the potential of JSI-124 for treating glioblastoma [15]. JSI-124 has been identified as a trigger for apoptosis in glioblastoma cells through the modulation of the JAK2/STAT3 signaling pathway. Specifically, JSI-124 can downregulate the levels of phosphorylated-STAT3, leading to apoptosis induction in glioblastoma cells. This mechanism highlights JSI-124 potential as a targeted therapeutic approach for glioblastoma treatment [16]. Furthermore, in addition to affecting the JAK2/STAT3 pathway, JSI-124 was also found to activate the NF-κB pathway to induce apoptosis in human glioblastoma cells [17]. JSI-124 activation of NF-κB presents an alternative mechanism through which it exerts pro-apoptotic effects, further highlighting its potential as a versatile therapeutic agent against glioblastoma. Studies have provided evidence that JSI-124 treatment effectively induces apoptosis in glioblastoma cells by activating the bcl-2 family proteins, which play a crucial role in promoting apoptosis and underscore JSI-124 potential as a glioblastoma-targeting therapeutic agent [18]. Furthermore, JSI-124 has been shown to promote apoptotic cell death in glioblastoma cells by downregulating the Aurora kinase family such as Aurora kinase A and B, as well as survivin, independent of seascape. These findings suggest that JSI-124 can effectively target multiple key regulators of cell survival and proliferation, making it a promising therapeutic approach for glioblastoma treatment [19]. Exposure to JSI-124 also leads to the downregulation of cyclin B1 and cdc2, resulting in cell cycle arrest in glioblastoma cells [18]. The multifaceted mechanisms by which JSI-124 exerts its anti-tumor activities include autophagy induction and cell cycle regulation. Additionally, JSI-124 has been found to inhibit critical cell-cycle-related proteins, such as survivin, Aurora kinase A, and Aurora kinase B. By inhibiting these proteins, JSI-124 disrupts the normal progression of the cell cycle, leading to defects and abnormalities. This interference with cell cycle progression represents a significant mechanism by which JSI-124 exerts its anti-tumor effects, providing potential therapeutic opportunities for conditions like glioblastoma [20]. Indeed, the multifaceted effects of JSI-124 on glioblastoma cells involve the activation of multiple signaling pathways. By targeting various molecular mechanisms, JSI-124 induces apoptosis and causes cell cycle arrest in glioblastoma cells. This wide range of actions underscores JSI-124 potential as a promising therapeutic agent for combating glioblastoma by simultaneously targeting multiple critical cellular processes involved in tumor growth and survival.

The exact mechanism by which JSI-124 inhibits glioblastoma and induces cell cycle arrest is not fully understood. Therefore, this study will investigate whether JSI-124 induces cell cycle arrest in glioblastoma cells and explore its specific molecular targets and pathways affecting cell cycle arrest. Experiments show that JSI-124 exhibits the ability to induce G2/M phase arrest, effectively arresting the cell cycle at a critical stage. This arrest is accompanied by the initiation of apoptosis due to caspase-3 activation, highlighting the dual mechanism of action of JSI-124. As our experiments demonstrate, these effects are implemented through regulation of the G2/M phase of the cell cycle and activation of caspase-3.

## 2. Materials and Methods

### 2.1. Reagents and Chemicals

We purchased Cucurbitacin I (CAS Number 2222-07-3) from Cayman Chemical and PrestoBlue™ from (ThermoFisher/Invitrogen, Carlsbad, CA, USA). Phosphate-buffered saline (PBS), dimethyl sulfoxide (DMSO), Trypan Blue Solution, Roswell Park Memorial Institute (RPMI) 1640 medium, Minimum Essential Medium (MEM), Trypsin-EDTA (0.25%) were purchased from Sigma (St Louis, MO, USA). Lastly, fetal bovine serum (FBS) and penicillin/streptomycin (P/S) were purchased from Gibco, the compound C (CC) from MedChemExpress (Monmouth Junction, NJ, USA), and the crystal violet solution from Sigma–Aldrich (St Louis, MO, USA).

### 2.2. Cell Culture

GBM8401 and U87MG cells were cultured using different media. GBM8401 cells were maintained in RPMI medium, while U87MG cells were cultured in MEM medium. Both medium contained 10% fetal bovine serum (FBS) and 1% penicillin/streptomycin (P/S) antibiotics (Thermo Fisher Scientific, Waltham, MA, USA). Both cell lines were maintained under standard incubation conditions of 37 °C and 5% CO_2_ to support optimal growth and viability. The culture medium and supplements provided necessary nutrients and antibiotics to support cell growth and prevent contamination during the procedures.

### 2.3. Cell Viability Analysis

Both GBM8401 and U87MG cells were seeded in a 96-well plate (3 × 10^3^ cells/well/200 μL) and incubated in an atmosphere containing 5% CO_2_, saturated humidity, and 37 °C for 24 h. The next day, cells were exposed to different concentrations of JSI-124 (0, DMSO, 0.5, 1, 2 μM) for 24–72 h. Cell viability after the JSI-124 (0, DMSO, 0.5, 1, 2 μM) treatment was measured using the MTT assay described by a previous study [12]. Cells and a new medium were added to the plate, and the absorbance was measured using a multi-well plate reader OD570 in Bio-Tek (Taipei, Taiwan).

### 2.4. Cell Cycle Assay

The cell cycle was detected using the FACSCalibur flow cytometry (FACSCalibur, BD, USA) [21]. GBM8401 and U87MG cells (3 × 10^3^ cells/well) were incubated in 6-well plates, in an atmosphere containing 5% CO_2_, saturated humidity, and 37 °C for 24 h. The next day, cells were treated with different concentrations of JSI-124 (0, DMSO, 0.5, 1 and 2 μM) for 24 h. Manufacturers’ protocols were followed for the staining with 500 μL PI solution (50 μg/ml PI, 100 μg/mL RNase A, 0.05% Triton X-100) and analysis of PI stain positive cells.

### 2.5. Mitotic Index Assay

We used MPM-2 to assess the mitotic index as previously described in the literature [22]. Cells treated with nocodazole were used as positive controls. Cells under the post-drug treatment underwent the following procedures: (1) fixation using 75% ethanol with storage at −20 °C for a minimum of 8 h, (2) staining using IFA-Tx buffer, (3) immunofluorescence staining using anti-mouse rabbit fluorescein isothiocyanate antibody (Serotec, Oxford, UK) and MPM-2 anti-phospho-Ser/Thr-Pro antibody, and (4) immunofluorescence analyzed using the FACSCalibur flow cytometer (BD Bioscience).

### 2.6. Apoptosis Assay

Apoptosis of JSI-124-treated cells was detected using Annexin V-FITC apoptosis dye, as previously described [23,24]. Dye-positive cells were observed and analysis was performed.

### 2.7. Caspase-3 Assay

Caspase-3 activity was detected using the caspase-3 FITC staining kit [25,26]. Cells were treated with JSI-124 (0, 0.5, 1, 2 µM) for 24 h. Cells were washed and stained with anti-caspase-3 antibody and were transferred from the centrifuge tubes.

### 2.8. Western Blotting

Western blotting was performed as previously described [27]. Then, the cells were collected, washed twice with PBST, and lysed with RIPA cell lysis reagent on ice for 30 min. After centrifugation, proteins were loaded with sodium dodecyl sulfate–polyacrylamide gel electrophoresis on 10% gels and were transferred onto polyvinylidene difluoride (PVDF) membranes (Millipore, Billerica, MA, USA) for 2 h. After washing, the membranes were incubated with primary antibodies (CCNB1; dilution 1:1000, CDK1; dilution 1:1000, CCNE1; dilution 1:1000) at 4 °C overnight. After washing the membranes three times, these were treated with secondary antibodies (dilution 1:20,000) at 37 °C for 1 h. After washing, antigens were detected with the Odyssey Near Infrared Fluorescence Imaging System (LI-COR). Using ImageJ software (NIH, Bethesda, MD, USA), the recorded values were normalized to β-actin (dilution 1:20,000) after densitometric analysis.

### 2.9. Next-Generation Sequencing (NGS)

Next-Generation Sequencing (NGS) was performed as previously described [28]. The original off-machine sequencing data first removed adapters and low-quality nucleic acid bases through fastp (v0.20.0). Raw off-board data and clean data were also evaluated using FastQC analysis. Then, SortMeRNA (v2.1b) was used to compare the reads with the reference rRNA database (Silva or Rfam) and further filter out the reads with rRNA in RNA-seq to obtain rRNA-free reads FASTQ file. The obtained FASTQ used HISAT2 (v2.1.0) for comparison with the reference genome. In the next step, the feature Counts (subread v2.0.1) was used to calculate the number of reads that were actually compared with the reference genome and output the performance scale table corresponding to each gene.

To identify significant KEGG categories, we performed the KEGG (https://www.genome.jp/kegg/, accessed on 6 December 2022) analysis to elucidate significantly the top 20 pathway maps. The horizontal axis shows the number of differentially expressed genes included in the pathway, the vertical axis shows the name of the KEGG pathway and the color indicates the degree of significance.

### 2.10. Statistics

The results were expressed as means ± SEM data from at least three independent experiments. The difference between groups was analyzed by GraphPad software (GraphPad, San Diego, CA, USA). One-way and two-way analyses of variance were used to calculate the statistical difference, and *p* < 0.05 was considered statistically significant.

## 3. Results

### 3.1. JSI-124 Suppressed the Growth of Malignant Glioma Cells by MTT

**Figure 2:**
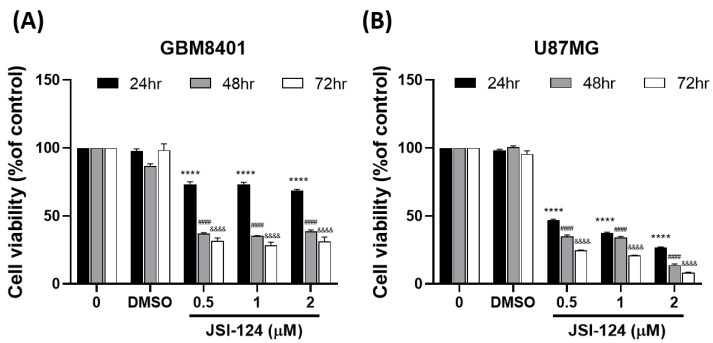
JSI-124 inhibited the cell viability in glioblastoma cells (**A**) GBM8401 and (**B**) U87MG cells. Cell viability was measured using the MTT reagent assay. The significance level was set at, **** *p* < 0.0001, &&&& *p* < 0.0001, #### *p* < 0.0001 signifying noteworthy distinctions between the control and treatment groups.

To assess the potential of JSI-124, both cell lines were exposed to different concentrations of JSI-124 (0, DMSO, 0.5, 1, 2 µM) for 24 h, 48 h, and 72 h. The MTT reagent assay was utilized to measure cell viability. These results demonstrated that the JSI-124 treatment significantly reduced the cell viability in human brain malignant glioma (GBM8401 cells) in a time-dependent manner. The U87MG cells experienced a dose- and time-dependent reduction in cell viability, suggesting JSI-124 selectivity as an anticancer agent against these cells. The IC50 values for JSI-124 in GBM8401 were estimated at 1.808 ± 1.03 μM (at 24 h), 1.1 ± 2.05 μM (at 48 h), and 0.4314 ± 1.89 μM (at 72 h) (Figure 2A). For U87MG cells, the IC50 values were determined to be 1.126 ± 0.52 μM (at 24 h), 1.723 ± 0.74 μM (at 48 h), and 1.441 ± 0.15 μM (at 72 h) (Figure 2B). These findings demonstrate a pronounced cytotoxic effect of JSI-124 specifically on GBM8401 and U87MG cells. Together, our findings show that JSI-124 significantly suppresses GBM growth.

### 3.2. JSI-124 Induced Apoptosis in Magilant Glioma Cells

**Figure 3:**
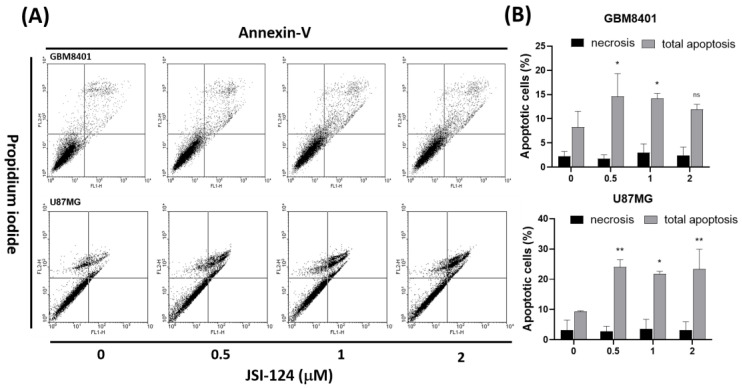
JSI-124 induced apoptosis in (**A**) GBM8401 and (**B**) U87MG cells. Cells were treated with JSI-124 (0, 0.5, 1, or 2 µM) for 24 h. Statistical significance was set at * *p* < 0.05, ** *p* < 0.01, ns *p* > 0.05.

To further validate the observed effects on apoptosis, we conducted an analysis of cell death utilizing PI staining and Annexin V-FITC. After treating cells with JSI-124 (0, 0.5, 1, 2 µM) for 4 h, cell death was evaluated, as depicted in Figure 3A. Flow cytometry was employed to quantify the percentage of cells undergoing apoptosis. The Annexin-FITC/PI assay demonstrated noteworthy changes in cell populations with and without JSI-124 treatment. Notably, the cells treated with JSI-124 exhibited a significant apoptosis compared with the untreated cells (Figure 3B). These findings provide compelling evidence that JSI-124 has the ability to induce apoptosis in glioblastoma cells, while necrosis was not observed as a significant outcome.

### 3.3. JSI-124 Promoted the Activity of Caspase-3 in GBM8401 and U87MG Cells

**Figure 4:**
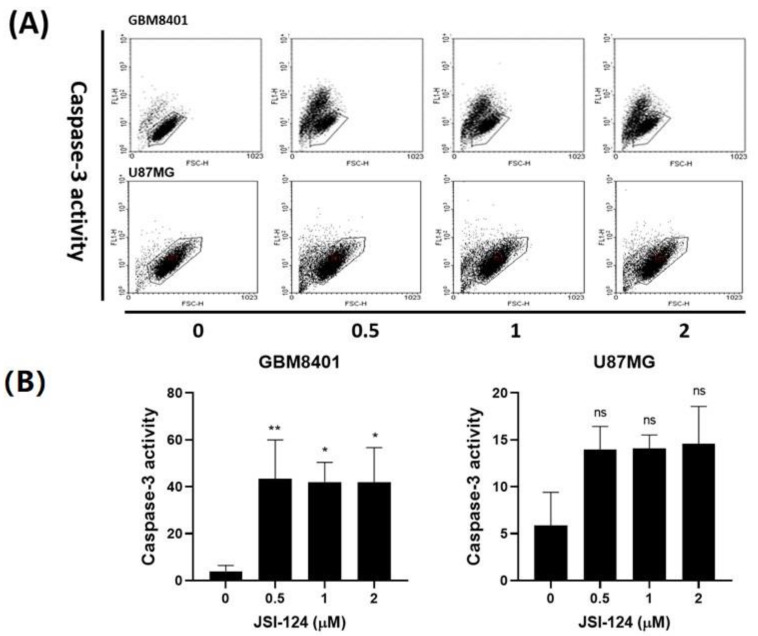
JSI-124 treatment increased the activity of caspase-3 in (**A**) GBM8401 and (**B**) U87MG cells. The results are presented as percentages relative to the control group, which was defined as 100%. Each data point represents mean ± S.D. from three independent experiments conducted in triplicates. Statistical significance was set at * *p* < 0.05, ** *p* < 0.01, ns *p* > 0.05.

Following the previous experiments, we conducted a caspase-3 assay to investigate the apoptosis induced by JSI-124 which occurred through caspase-3-dependent apoptosis. The JSI-124 was varied (0, 0.5, 1, 2 µM), and it was observed that, as the JSI-124 concentration increased, there was a corresponding increase in the number of cells positive for active caspase-3. Importantly, the caspase-3 was detected in significant quantities in both JSI-124-treated cell types, as illustrated in Figure 4A. These results strongly support the notion that JSI-124 induces cell death in GBM8401 and U87MG cells through caspase-3-dependent apoptosis, although this was not significant in U87MG cells. Collectively, our findings provide compelling evidence that the reduced viability of glioma cells induced by JSI-124 can be attributed to its ability to induce apoptosis in a caspase-dependent manner.

### 3.4. JSI-124 Induced G2/M Arrest in Glioblastoma Cells

**Figure 5:**
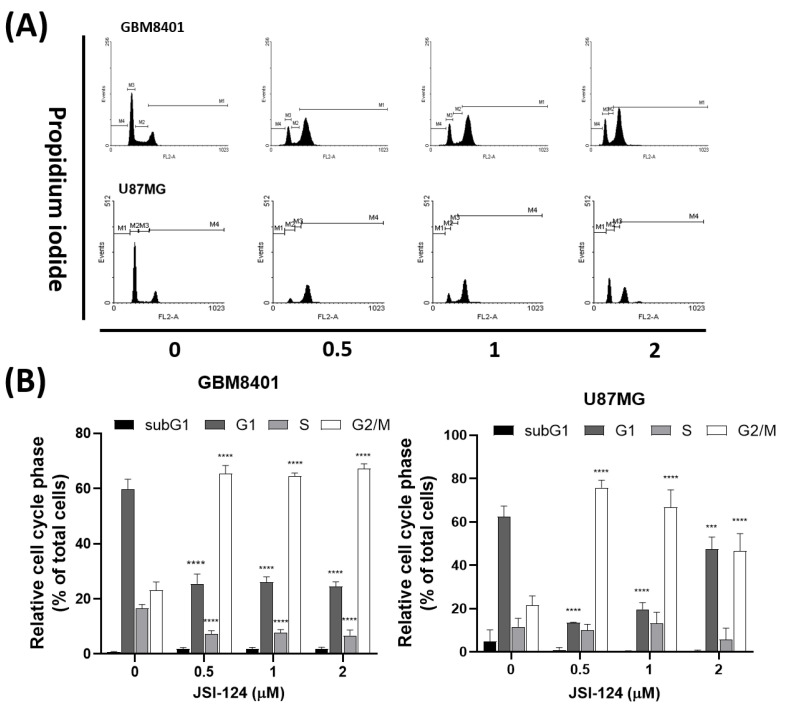
(**A**) Cell cycle analysis to assess the effect of the JSI-124 treatment on cell cycle progression in glioblastoma cells (GBM8401 and U87MG cells). (**B**) DNA content analysis was performed, followed by quantification using flow cytometry. Statistical significance was set at *** *p* < 0.05, **** *p* < 0.0001.

To examine how JSI-124 influences the advancement of the cell cycle in glioma cells, we executed experiments following the procedure outlined in Figure 5. Glioma cells were subjected to various concentrations of JSI-124 (0, 0.5, 1, 2 µM) for a specific duration, and subsequently analyzed through PI staining to determine the distribution of cells across the cell cycle. The findings unveiled an increase in the percentage of cells at the G2 phase and M phase subsequent to JSI-124 treatment.

### 3.5. JSI-124 Causes Mitotic Arrest in Glioblastoma Cells

**Figure 6:**
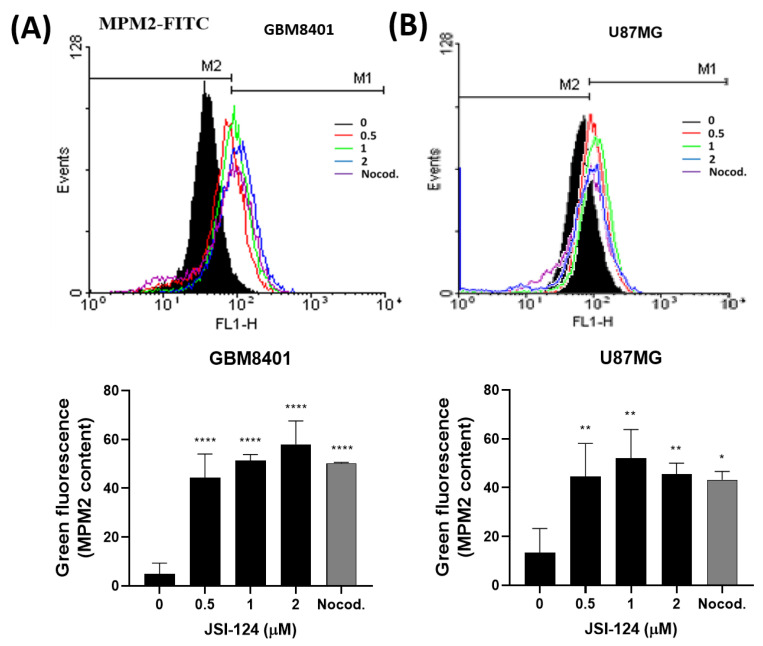
Flow cytometry analysis was conducted after staining with MPM-2 and PI in (**A**) GBM8401 cells and (**B**) U87MG cells. Quantification of MPM-2 content showed an increase in mitotic activity in glioblastoma cells upon treatment with JSI-124. Statical significant was set at * *p* < 0.05, ** *p* < 0.01, **** *p* < 0.0001.

We performed MPM-2 staining to understand the mechanism behind cell cycle arrest, as illustrated in Figure 6. The JSI-124 treatment led to elevated MPM-2 staining, suggesting the initiation of mitotic arrest. We treated cells with nocodazole (10 μg/mL) as a positive control, nocodazole being a known inducer of metaphase arrest. The findings revealed presence of an elevated count of G2/M phase cells in both cell lines after the JSI-124 treatment, as depicted in Figure 5. Additionally, the JSI-124 treatment enhanced protein synthesis during mitosis, as illustrated in Figure 6. To sum up, these results imply that JSI-124 stimulates DNA synthesis, prompting cells to transition to the G2 phase but not to the M phase, ultimately resulting in mitotic arrest.

### 3.6. JSI-124 Regulates Cell-Cycle-Related Proteins to Induce Cell Cycle Arrest

**Figure 7:**
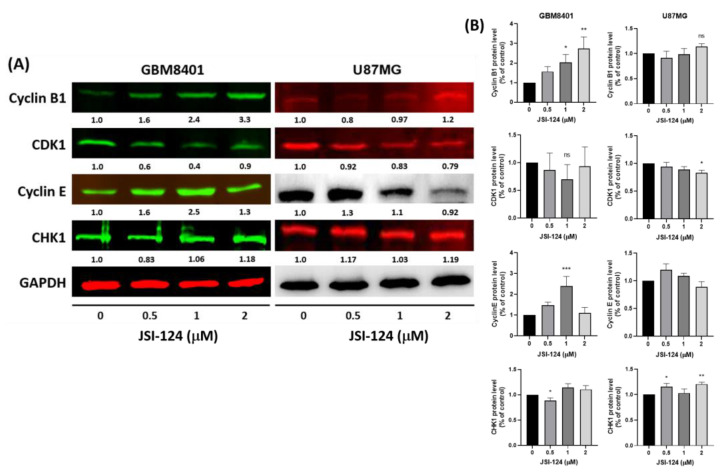
The effect of JSI-124 on glioma cells, inducing cell cycle arrest. (**A**) We examined the influence of JSI-124 on the expression of cell-cycle-related proteins by subjecting the cells to JSI-124 treatment. Specifically, we assessed the levels of CHK1, CDK1, cyclin B1, and cyclin E. Western blotting was employed to detect the expression of these cell-cycle-related proteins. (**B**) Quantification of JSI-124 regulatory protein expression (GAPDH as loading controls). Statistical significance was set at * *p* < 0.05, ** *p* < 0.01, *** *p* < 0.001. Western blot original data are shown in Supplementary Materials.

How does JSI-124 exerts its effects on GBM8401 and U87MG cells? The expression levels of different cell-cycle-related proteins were examined using Western blot analysis, as illustrated in Figure 7A. These results demonstrate that treatment with JSI-124 led to a decrease in CDK1 expression, while the expression levels of CHK1, CCNB1, and CCNE1 increased, as illustrated in Figure 7B. These results suggest that JSI-124 treatment modulates the cell-cycle-related proteins and genes, potentially contributing to the observed cell cycle arrest and mitotic arrest in GBM8401 and U87MG cells.

### 3.7. Genes and Genomes Enrichment Analysis Were Performed after JSI-124 Treatment in Glioma Cells

**Figure 8:**
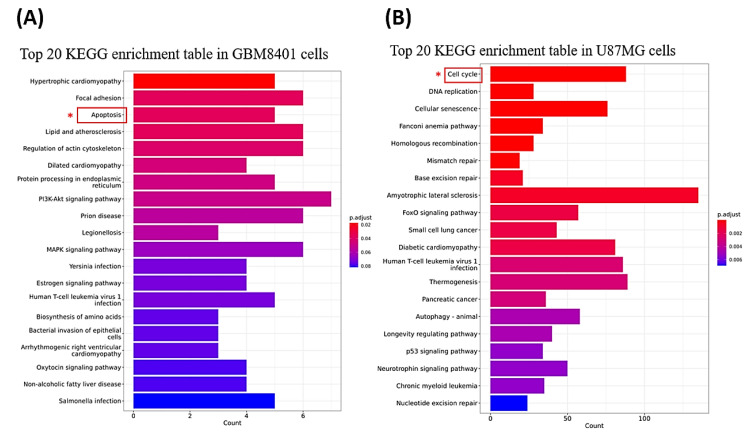
The genes and genomes enrichment analysis after JSI-124 treatment in GBM8401 (**A**) and U87MG cells (**B**). * We found that JSI-124 may affect U87MG cells by regulating the cell cycle pathway and GBM8401 cells by regulating the apoptosis pathway.

We performed genes and genomes enrichment analysis using KEGG (Kyoto Encyclopedia of Genes and Genomes) analysis on cells treated with 2 μM of JSI-124. The significantly enriched KEGG pathways in each cell line are presented in Figure 8A,B. Notably, the gene expression profiles of U87MG and GBM8401 cells were significantly different after JSI-124 treatment. In GBM8401 cells, we observed significant regulation of apoptosis-related genes, suggesting that JSI-124 activated or affected the apoptotic signaling pathway in these cells. This finding suggests that apoptosis may be a key mechanism of action of JSI-124 in GBM8401 cells, which may contribute to their anticancer properties (Figure 8A). On the other hand, in U87MG cells, we observed significant regulation of cell-cycle-related genes, suggesting that JSI-124 activated or affected the cell cycle signaling pathway in these cells (Figure 8B). This finding suggests that JSI-124 may have an effect on U87MG cells by regulating cell cycle progression. The cell cycle arrest and mitotic arrest observed in U87MG cells, as previously described, are consistent with this finding and suggest that regulation of cell-cycle-related pathways may contribute to the therapeutic potential of JSI-124 in U87MG cells. These results highlight the cell-type-specific effects of JSI-124 treatment on gene expression and signaling pathways and emphasize the need for further investigation to fully understand the exact mechanisms through which JSI-124 exerts its anticancer effects on different glioma cell lines.

### 3.8. Differential Expression of Apoptosis and G2/M Cell-Cycle-Relative Signaling Gene in Glioma Cells via NGS Analysis

**Figure 9:**
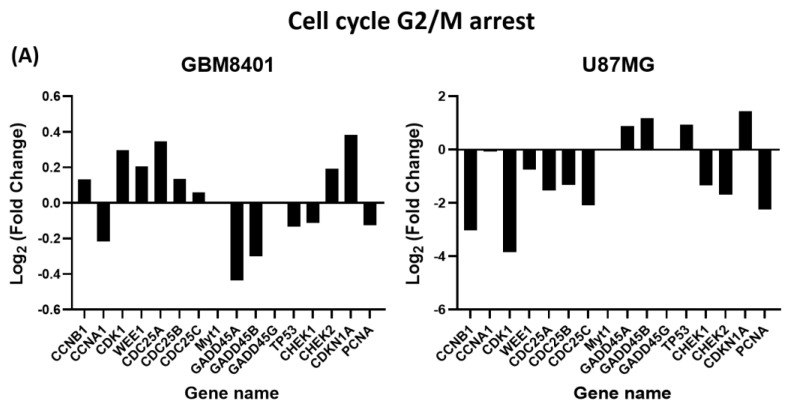

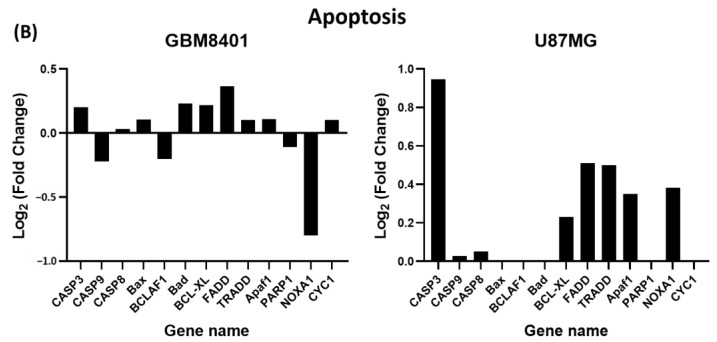
JSI-124-regulated cell cycle and apoptosis-related mRNA expression in GBM8401 and U87MG cells. Exposure to 8 µM of JSI-124 for 24 h up- or downregulates G2/M phase-related genes expression (**A**) and apoptosis-related genes expression (**B**).

Our results further confirm that JSI-124 treatment induces apoptosis in mRNA genes and G2/M cell-cycle-related mRNA genes in GBM cells, as shown in Figure 9A,B. To analyze the mRNA expression, the levels of mRNA of JSI-124-treated and vehicle-treated groups were compared. Genes showing ploidy changes greater than two were considered significantly upregulated, while genes showing ploidy changes lower than two were considered downregulated. We identified a total of 29 genes that were significantly altered in JSI-124-treated cells, with higher Z values than in the blank control group. In these genes, 13 were associated with apoptosis, while 16 were associated with cell cycle G2/M arrest. These findings provide valuable insights into the molecular mechanisms behind JSI-124 effects on glioma cells, highlighting specific genes and pathways that may contribute to their apoptotic and cell cycle regulatory properties.

## 4. Discussion

Based on our results, JSI-124 may play a role in the apoptosis of glioblastoma cells and regulate the cell cycle pression, suggesting its potential as a novel therapeutic target drug. Firstly, JSI-124 inhibits cell viability through a caspase-3-dependent apoptotic pathway. Furthermore, JSI-124 has the potential to trigger G2/M cell cycle arrest. Additionally, JSI-124 treatment can regulate mitotic arrest in the cell cycle, promoting DNA synthesis to the G2 phase. Finally, NGS analysis revealed that JSI-124 regulates genes associated with apoptosis and the cell cycle, thereby influencing apoptosis and cell cycle arrest, affecting, in turn, cell progression.

The overactivation of the JAK/STAT pathway is frequently observed in cancer and is associated with poor prognosis [29]. Therefore, it is reasonable to speculate that JSI-124 may exert its anticancer effects by inhibiting the JAK2/STAT3 signaling pathway. Current research indicates that the JAK/STAT signaling pathway is implicated in the development of various cancer types, including glioblastoma [30,31,32]. JSI-124 has been identified as a selective inhibitor of the JAK2/STAT3 signaling pathway in various types of cancer [14,20,33]. Previous investigations have provided evidence that JSI-124 possesses the capability to hinder the JAK/STAT3 pathway across different cancer categories, including glioblastoma. Moreover, JSI-124 exhibits the capacity to restrain the proliferative behavior of glioblastoma cells by focusing on the consistently active STAT3 signaling route [18]. In addition, our experimental outcomes highlight JSI-124 potential to regulate genes and proteins linked to apoptosis as well as the cell cycle. This regulation promotes apoptosis and triggers cell cycle arrest, thereby impeding further progression. In conclusion, these revelations point towards JSI-124 potential as a promising anticancer agent for glioblastoma.

Apoptosis can be triggered by either the extrinsic pathway or the intrinsic pathway [34]. Previous research has reported that JSI-124 induces apoptosis, a phenomenon observed via Annexin V/PI and TUNEL detection in various cancer cells [13,16,18]. However, some natural compounds, such as alkaloid trigonelline, have been discovered to enhance the susceptibility of cancer cells to apoptosis. This finding highlights the potential of certain natural compounds to effectively inhibit and promote programmed cell death in cancer cells [35,36]. In this study, it was found that cucurbitacin I (JSI-124) induces cytotoxicity in glioblastoma cells. The degree of apoptosis increases with dosage and time, as evidenced by the escalating number of Annexin-V-positive cells after JSI-124 incubation. Additionally, the activity of caspase-3 increased, indicating that JSI-124 induces apoptosis in glioblastoma cells through a caspase-3-dependent mechanism, thus promoting intrinsic apoptosis. Consequently, it can be concluded that JSI-124 exhibits potential as a selective anticancer agent by inducing caspase-3-dependent apoptosis in cells.

The cell cycle is supervised by four checkpoints, while the distinctive feature of cancer cells is their uncontrolled proliferation [13]. However, cancer cells lose control over these checkpoints, resulting in unrestrained growth. Strengthening the surveillance of cell cycle checkpoints has emerged as a strategy in the development of anticancer drugs. These therapeutic agents often target cell cycle checkpoints to curtail the uncontrolled growth observed in cancer cells. Previous studies have demonstrated that cucurbitacin I (JSI-124) stimulates programmed cell death, such as apoptosis, across various tumor types [13,18]. Nevertheless, further investigation is needed to determine its potential in modulating cell cycle checkpoint surveillance. Several prior studies have explored JSI-124 impact on the cell cycle, but the precise mechanisms underpinning its ability to induce cell cycle arrest remain uncertain. We found that JSI-124 induced cell cycle arrest at G2/M. During the cell cycle, the early G1 phase is distinguished by the stimulation of mitogens, which triggers the expression of cyclin D. This cyclin D activation subsequently activates CDK4/6. As G1 progresses into its later stage, the levels of cyclin E and A increase. The transition from G1 to the S phase is facilitated by CDK2, which forms complexes with cyclins E and A. In order to progress into the G2 phase and exit the M phase, cyclin B binds and activates CDC2, which plays a crucial role in these transitions [37]. Therefore, there is reason to believe that the G2/M arrest observed in JSI-124-treated GBM cells is partly due to the regulated expression of cyclin B and CDC2.

Although the exact mechanisms underlying the anticancer effects of cucurbitacin I (JSI-124) remain incompletely understood, recent research has shown that JSI-124 can specifically induce G2/M cell cycle arrest, as illustrated in Figure 4 and Figure 5. The cell cycle is controlled by a complex network of regulators [38]. Positive regulators facilitate progression through various checkpoints, including cyclins and cyclin-dependent kinases (CDKs) [39]. Conversely, negative regulators act as brakes on the cell cycle, and these include p53, Rb, p21, CHK1, and CHK2 [38]. Although these results suggest that JSI-124 enhances the ability of the G2/M checkpoint and interferes with protein synthesis during mitosis, the exact mechanisms remain to be fully elucidated. Additionally, research has indicated that JSI-124 can modulate positive cell cycle regulators such as cyclins and CDKs and even inhibit cyclin/CDK complexes. This suggests that JSI-124 may induce cell cycle arrest through this mechanism. Furthermore, JSI-124 can promote negative cell cycle regulators and suppress positive cell cycle regulators, as illustrated in Figure 7. After JSI-124 treatment, there is a downregulation of cyclins and CDKs and an upregulation of checkpoint kinases such as CHK1, ultimately resulting in cell cycle arrest.

This study has several limitations. Firstly, it is important to note that this research is primarily based on in vitro experiments, and it should be acknowledged that this study did not progress to in vivo experiments, organoid models, or proteotranscriptomic analysis. Therefore, the design of animal experiments and human trials should be considered as a continuation of this research. Additionally, culturing and studying microglia cells come with some limitations and challenges. Some key restrictions include the difficulty associated with the maintenance of microglia cells, challenges in long-term cultivation, and the control of their activation state. Therefore, culturing and studying microglia cells are complex tasks that require specialized knowledge and experimental skills.

**Figure 10:**
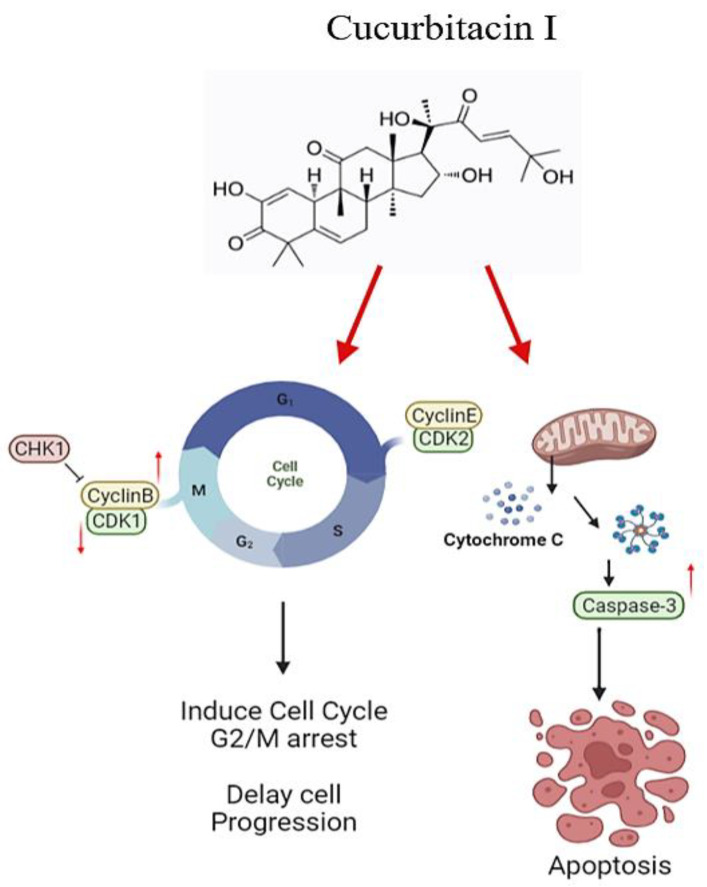
JSI-124 treatment of GBM cells induces apoptosis and cell cycle arrest. We found that JSI-124 involves cell cycle regulators and apoptosis mechanisms, thereby inhibiting cell growth progression. The cell cycle comprises several phases, including G1, S, G2, and M phases. The figure was created using BioRender.com.

Anticancer therapies encompass two primary strategies, one of which involves inducing cell cycle arrest in tumor cells, while the other entails inducing apoptosis. As illustrated in Figure 10, JSI-124, a potential anticancer drug with a dual function, indeed exhibits promising anticancer capabilities in cellular experiments by promoting apoptosis and causing cell cycle arrest. This results in the halting of the cell cycle progression. Consequently, JSI-124 holds the potential to serve as an innovative therapeutic agent capable of triggering both apoptosis and cell cycle arrest. 

## 5. Conclusions

In conclusion, our study demonstrates that JSI-124 inhibits cell viability through a caspase-3-dependent apoptotic pathway and induces G2/M cell cycle arrest. Additionally, JSI-124 treatment can regulate mitotic arrest in the cell cycle. Finally, NGS analysis revealed that JSI-124 regulates apoptosis and cell-cycle-related genes, thereby influencing apoptosis and cell cycle arrest, consequently affecting cell progression. This renders JSI-124 a promising candidate for the development of novel anticancer drugs.

## Data Availability

Data are available from the corresponding author upon reasonable request and with approval from “I-Shou University School of Medicine, Taiwan”.

## Supplementary Materials

The following supporting information can be downloaded at: https://www.mdpi.com/article/10.3390/biomedicines11112999/s1, Figure S1: western blot original data.
