# Supplementary material for: JSI-124 Induces Cell Cycle Arrest and Regulates the Apoptosis in Glioblastoma Cells
Source: Biomedicines. 2023 Nov 8;11(11):2999. doi: 10.3390/biomedicines11112999 (PMC10669163; doi:10.3390/biomedicines11112999)
Supplement: Supplementary file 1 [file biomedicines-11-02999-s001.zip › biomedicines-2655041-supplementary.pdf]

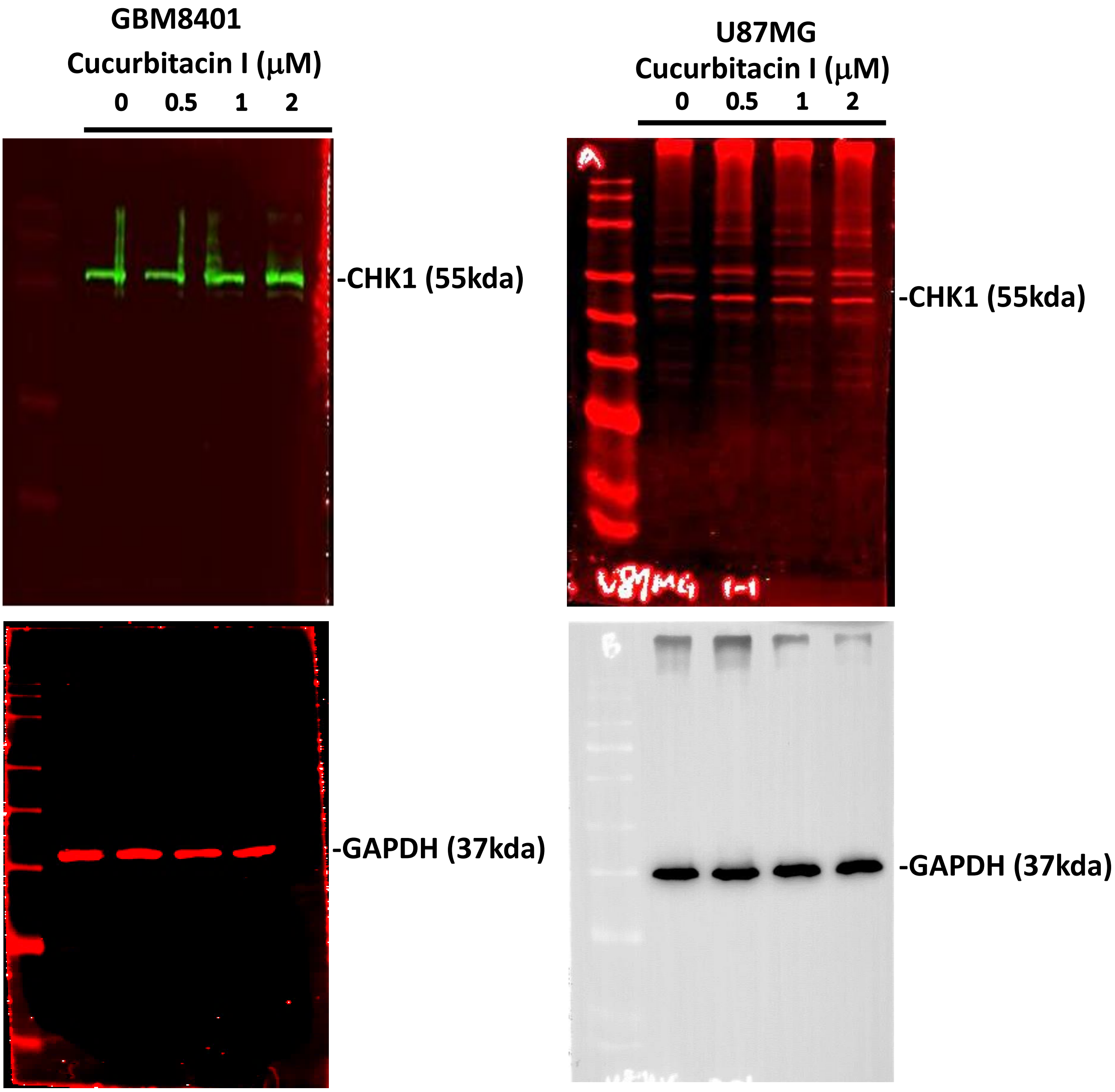
GBM8401
Cucurbitacin I (μM)
0 0.5 1 2
-CHK1 (55kda)
-GAPDH (37kda)
U87MG
Cucurbitacin I (μM)
0 0.5 1 2
-CHK1 (55kda)
-GAPDH (37kda)

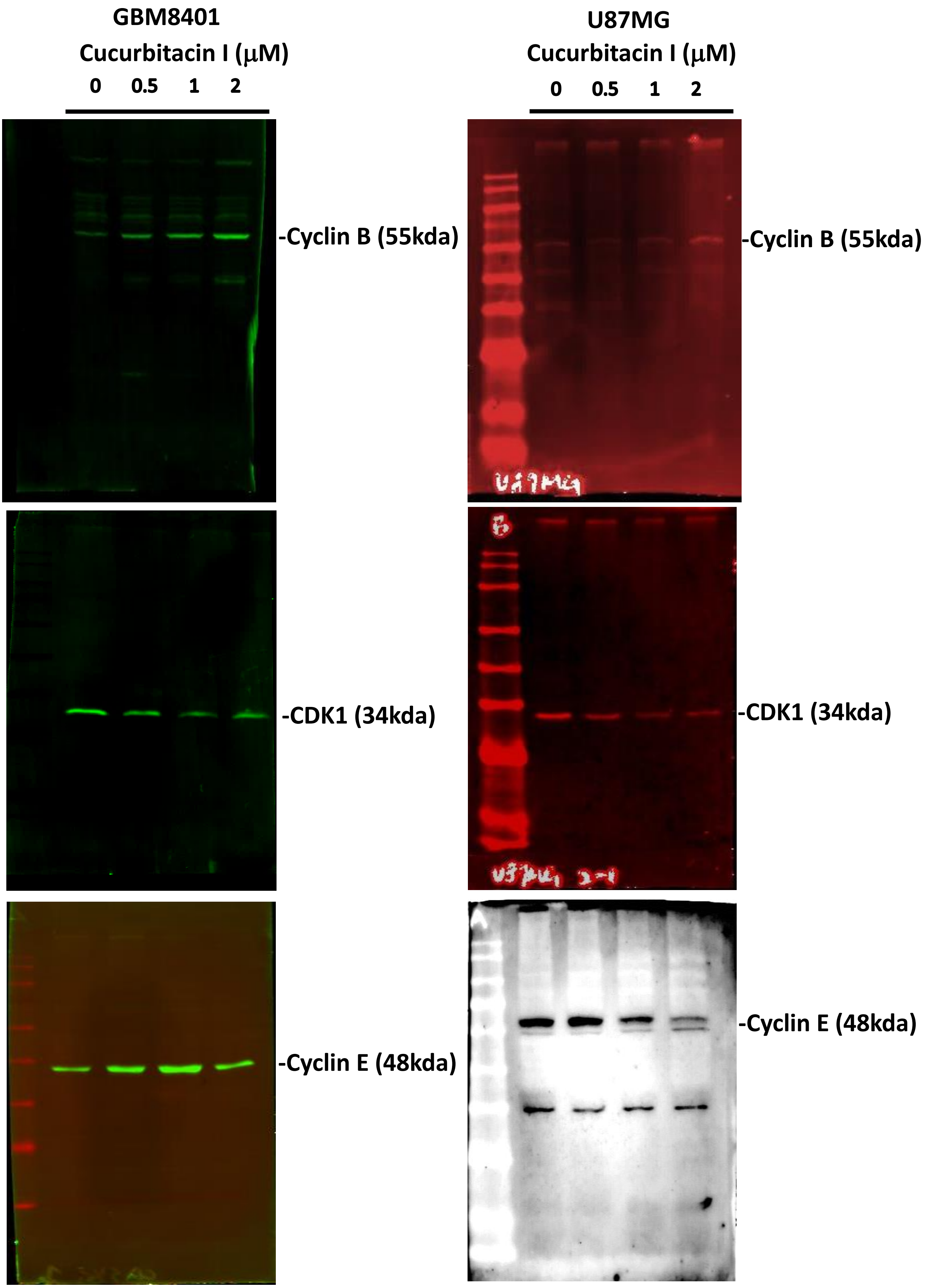

Figure S1: western blot original data.
